# Predictors of right ventricular failure after left ventricular assist device implantation

**DOI:** 10.3325/cmj.2014.55.587

**Published:** 2014-12

**Authors:** Marijan Koprivanac, Marta Kelava, Franjo Sirić, Vincent B. Cruz, Nader Moazami, Tomislav Mihaljević

**Affiliations:** 1 Heart and Vascular Institute, Department of Thoracic and Cardiovascular Surgery, Cleveland Clinic, Abu Dhabi, United Arab Emirates; 2 Anesthesiology Institute, Cleveland Clinic, Cleveland, OH, USA; 3 Division of Cardiothoracic Surgery, University of Alabama at Birmingham, Birmingham, AL, USA; 4 Cleveland Clinic Lerner College of Medicine of Case Western Reserve University School of Medicine, Cleveland, OH, USA

## Abstract

Number of left ventricular assist device (LVAD) implantations increases every year, particularly LVADs for destination therapy (DT). Right ventricular failure (RVF) has been recognized as a serious complication of LVAD implantation. Reported incidence of RVF after LVAD ranges from 6% to 44%, varying mostly due to differences in RVF definition, different types of LVADs, and differences in patient populations included in studies. RVF complicating LVAD implantation is associated with worse postoperative mortality and morbidity including worse end-organ function, longer hospital length of stay, and lower success of bridge to transplant (BTT) therapy. Importance of RVF and its predictors in a setting of LVAD implantation has been recognized early, as evidenced by abundant number of attempts to identify independent risk factors and develop RVF predictor scores with a common purpose to improve patient selection and outcomes by recognizing potential need for biventricular assist device (BiVAD) at the time of LVAD implantation. The aim of this article is to review and summarize current body of knowledge on risk factors and prediction scores of RVF after LVAD implantation. Despite abundance of studies and proposed risk scores for RVF following LVAD, certain common limitations make their implementation and clinical usefulness questionable. Regardless, value of these studies lies in providing information on potential key predictors for RVF that can be taken into account in clinical decision making. Further investigation of current predictors and existing scores as well as new studies involving larger patient populations and more sophisticated statistical prediction models are necessary. Additionally, a short description of our empirical institutional approach to management of RVF following LVAD implantation is provided.

Heart failure is one of the most common causes of death in western world. Number of patients diagnosed with heart failure is growing and it remains a target for many preventive and treatment efforts (1,2). Mechanical circulatory support (MCS) devices, including ventricular assist devices (VADs) represent an important treatment modality. Given common etiology of end stage heart failure, left ventricular assist devices (LVADs) are of greater interest. Right ventricular assist devices (RVADs) are reserved for situations in which right ventricular failure (RVF) develops, usually as pathophysiological sequels of left ventricular failure (LVF) or LVAD implantation (Figure 1 and 2).

**Figure 1:**
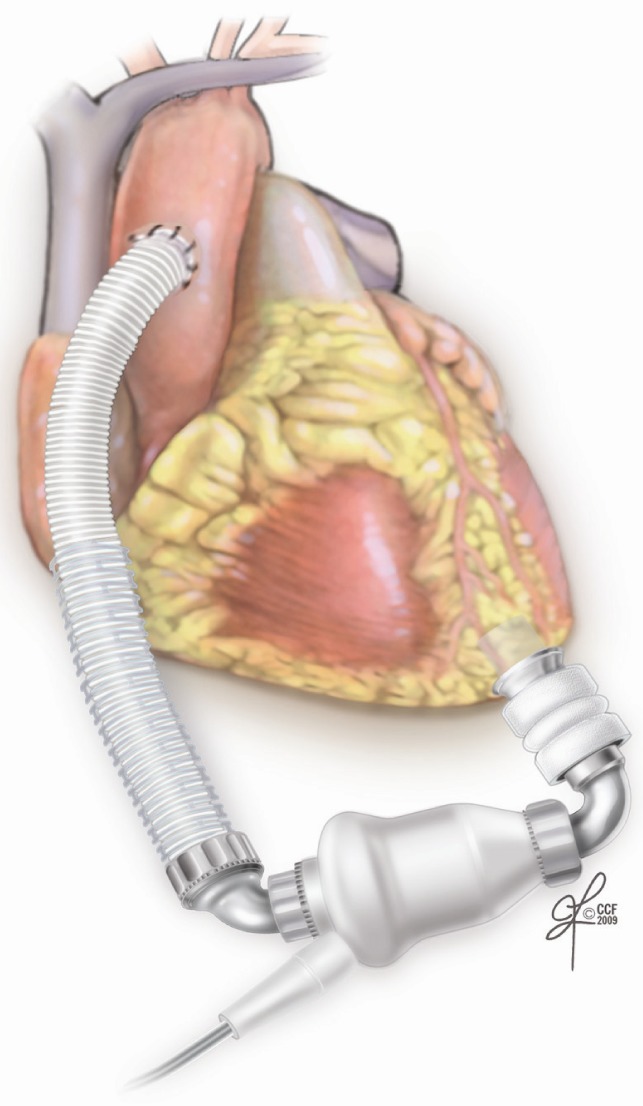
Illustration of Heart Mate II (Thoratec, Pleasanton, CA, USA) implantation. Reprinted with permission, Cleveland Clinic Center for Medical Art & Photography ©2002. All Rights Reserved.

**Figure 2:**
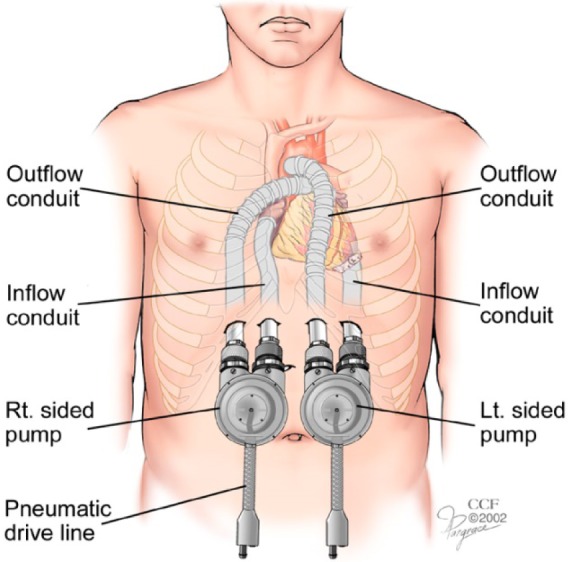
Illustration of Thoratec PVAD (Thoratec, Pleasanton, CA, USA) (left ventricular assist device [LVAD], right ventricular assist device [RVAD], and biventricular assist device [BIVAD]). Reprinted with permission, Cleveland Clinic Center for Medical Art & Photography ©2002. All Rights Reserved.

Indications for LVAD implantation include: 1. bridge to transplantation (BTT); 2. bridge to candidacy for transplantation; 3. destination therapy (DT), ie, patients who are not suitable for transplantation; and 4. temporary support for patients whose cardiac function is expected to recover.

Scientific and technological improvements of LVAD implantation, as well as perioperative medical management have led to significant reduction in complication rates, and improved survival and quality of life, all leading to outcomes approaching those achieved with heart transplant (3-8). Therefore, it is not surprising that overall rate of LVAD implantation increases every year, especially the number of LVADs for destination therapy (9) (Figure 3).

**Figure 3:**
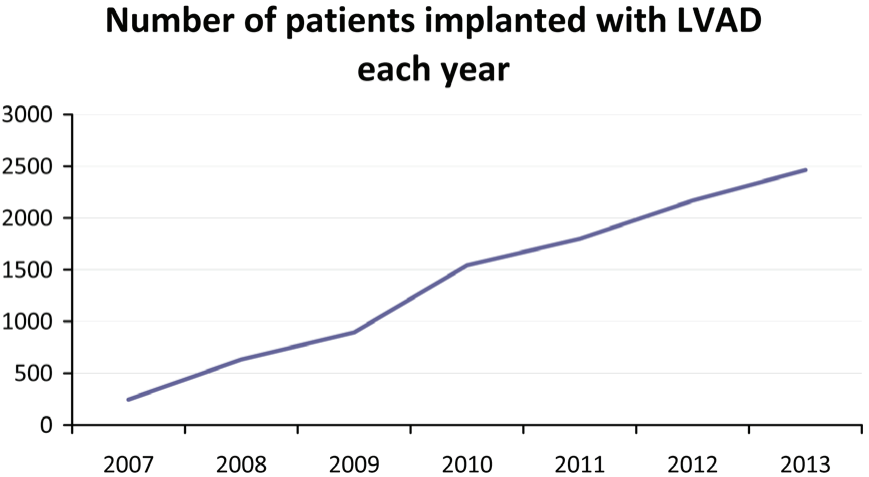
Trend of left ventricular assist device (LVAD) implantations in last seven years, per INTERMACS Quarterly Statistical Report, 1st Quarter, 2014 (*https://www.uab.edu/medicine/intermacs/images/CMS/CMS_Report_2012_Q4.pdf*)

RVF has been recognized as a serious complication of LVAD implantation, and as such has been a subject of considerable research. The purpose of this article is to review existing literature on predictors and risk scores for RVF following LVAD implantation. To identify studies of interest we used MEDLINE search with Boolean term “AND,” limited to Title/Abstract and English language using terms “RV failure,” “LVAD,” and “predictors,” which yielded 15 articles. Citation indexing on the included studies was done to screen for other relevant publications not identified by our search strategy, which yielded additional 40 articles that were reviewed in detail. After careful review of these 55 articles, we identified a total of 11 studies that developed original prediction models for right ventricular failure after LVAD. Of these 11 studies, 7 used available preoperative and intraoperative demographic, laboratory, and comorbidity variables in their models (Supplementary Table 1)(web extra material 1). The remaining 4 studies used echocardiographic variables (Supplementary Table 2)(web extra material 2).

## Importance of right ventricular failure after left ventricular device implantation

Many definitions of RVF after LVAD have been used. Most common definition describes RVF after LVAD as need for intravenous inotrope or pulmonary vasodilator therapy for 14 days postoperatively and/or need for RVAD implantation (10-14). A few studies defined RVF as requirement for RVAD only (15,16), whereas others used two or more of the following hemodynamic parameters to define RVF; central venous pressure greater than 16 mm Hg, mean arterial pressure lower than 55 mm Hg, cardiac index lesser than 2 L/min/m2, inotropic support more than 20 units, mixed venous saturation lower than 55%, all in the absence of cardiac tamponade (17).

Reported incidence of RVF after LVAD varies from 6% to 44% (10-12,14,16,18-33). This is mostly due to differences in definitions of RVF, different types of LVADs (continuous vs pulsatile), and differences in patient populations.

Patients who develop RVF have higher incidence of end organ dysfunction, and overall worse morbidity and mortality. In addition, RVAD implantation as a treatment option for RVF leads to increased morbidity secondary to increased infection risk, need for transfusion, and risk of device failure. Therefore, one of the most important issues since introduction of LVAD as treatment for heart failure has been to determine the need and optimal timing of RVAD support.

There have been many studies that developed prediction models and scores for RVF risk after LVAD, with common purpose to improve patient selection, and successfully predict the need for BiVAD at initial surgery, possibly leading to improved outcomes.

Although it seems intuitive that implanting BiVAD early during initial surgery would improve outcomes in patients who are at risk for RVF, and thereby prevent end organ dysfunction and associated complications, supporting data are lacking. To the best of our knowledge, there have been only two studies showing outcome benefit for planed vs delayed RVAD implantation, while others showed either no benefit or mixed results (15,16,28,34). However, it is important to note that these were all retrospective studies with smaller number of patients, and further research is necessary to provide more accurate evidence. Clinical expertise and judgment will continue to guide decision making until more evidence becomes available.

## Pathophysiology and proposed mechanisms of right ventricular failure

Factors determining right ventricular (RV) output and function include RV preload, afterload, and contractility. Changes in physiology following LVAD affect all these RV output determinants and may lead to acute RV failure.

RV preload increases as a result of increased left ventricular (LV) output which might increase up to 100% following LVAD implantation (35). In addition, patients often receive substantial amount of fluids and blood products during perioperative period. This acute increase in preload leads to overstretching of cardiac myofibrils beyond the point of optimal contractility based on Frank Starling principle, and decreased RV stroke volume. Furthermore, increase in RV preload may lead to RV annular dilatation and tricuspid regurgitation adding to RV demand.

It is well known that the most common cause of RVF is LV failure (36). Left ventricular end diastolic pressure (LVEDP) is transmitted into increased pulmonary pressure leading to increased RV afterload and pulmonary hypertension (PHTN). LVAD implantation directly influences this mechanism, leading to decreases in RV afterload and in many cases resolution of PHTN (17,37,38). The time frame in which this occurs is variable. It is important to note that PHTN resolution is not universal, such as in cases of primary PHTN and PHTN related to pulmonary disease, as well as when pulmonary vasculature remodeling has already taken place.

Contractility of RV is influenced by LV function through the concept of ventricular interdependence. Effect of ventricular interdependence is most prominent in a setting of loading changes such as after LVAD implantation, and has an important role in pathophysiology of RV dysfunction following LVAD (39). It has been suggested that substantial amount of RV systolic pressure and stroke volume results from LV contraction, and that interventricular septum plays the most important role (40). Structural organization and direction of myofibrils in the interventricular septum resembles more closely to the structure of left ventricular free wall where fibers are arranged in an oblique fashion as opposed to right ventricle, where mostly transverse and longitudinal fiber orientation is found. As septum contracts it contributes to contraction effort of RV free wall and also serves as its support structure. The dependence of RV output to interventricular septum and LV contractility has been proven in many animal models (41-43).

## Factors predicting right ventricular failure following left ventricular device implantation

RVF complicating LVAD implantation is associated with worse postoperative mortality and morbidity including worse end-organ function, longer hospital length of stay, and lower success of BTT therapy (10,11,15,27). Importance of RVF and its predictors in a setting of LVAD implantation has been recognized early, as evidenced by abundant number of attempts to identify independent risk factors and develop RVF predictor scores. However, most of available literature are retrospective studies from single institutions that have similar limitations, including small sample size, lack of score validation, and for some earlier studies inclusion of patients receiving pulsatile LVADs only. Additional factors making evaluation and comparison of different predictor models and respective risk scores difficult is poor consistency in definition of RVF outcome, heterogeneity of variables considered in construction of prediction models, and variability in inclusion of BTT and DT patients. Multivariate regression analysis was used to identify predictors in all studies except one, which employed a novel decision tree prediction model. Models also differ in determination of multivariate analysis endpoint, with some focusing on RVF and others on mortality.

As already indicated, available prediction models differ with respect to inclusion of different types of LVADs. The next few sections provide a summary of most important pertinent studies in a chronological order, starting with those involving patients receiving pulsatile flow LVADs and moving toward combination cohorts and newer generation continuous LVADs cohorts only.

Destination therapy risk score (DTRS) was based on analysis of a cohort that included 222 patients receiving pulsatile flow LVADs for DT (44). Variables of interest included demographic characteristics, cardiac and noncardiac comorbidities, hemodynamic and laboratory parameters. Model was developed to predict 90-day in-hospital mortality (N = 27%), rather than RV failure directly, and identified platelet count ≤148 × 10^3^/mcgL, serum albumin ≤3.3 g/dL, international normalization ratio (INR)>1.1, vasodilator therapy at time of implantation, mean pulmonary artery pressure ≤25.3 mm Hg, aspartate aminotransferase >45 U/dL, hematocrit <34%, blood urea nitrogen >51 U/dL, and lack of intravenous inotropic support as statistically significant predictors. A weighted risk score was assigned to each of the above nine variables to develop DTRS. Discrimination C-statistic for DTRS was good (C-statistic value 0.89; sensitivity, 82.6%; and specificity, 80.0% at *P* = 0.24). Patients were divided into 4 operative risk categories for 90-day in-hospital mortality. The observed survival to hospital discharge in low-, medium-, high-, and very high-risk operative candidates was 87.5%, 70.5%, 26%, and 13.7%, and 1-year survival was 81.2%, 62.4%, 27.8%, and 10.7%, respectively. Although this was one of the most commonly used scores clinically, its utility is questionable in today’s era of newer generation continuous flow LVADs predominance. A study evaluating DTRS for continuous LVADs found that the score was able to discriminate statistically significant differences in survival only for DT, and not BTT patients, but with poor discrimination in both groups (C-statistic 0.54 and 0.58, for DT and BTT respectively) (45) ·

Another earlier model developed based on analysis of pulsatile flow LVAD data from University of Pennsylvania (28) included 266 patients and looked at a total of 36 demographic, clinical, hemodynamic, and laboratory parameters. The endpoint was RVF, defined as a need for RVAD placement, and the incidence was 37%. In this series, multivariate analysis identified RV stroke work index ≤0.25 mm Hg × L/m^2^ (odds ratio [OR] 5.1), severe pre-operative RV dysfunction (OR 5.0), pre-operative creatinine ≥1.9 mg/dL (OR 4.8), previous cardiac surgery (OR 4.5), and systolic blood pressure ≤96 mm Hg (OR 2.9) as important predictors for RVF. Authors emphasized severe preoperative RV failure as likely the most significant factor determining which patients would require BiVAD, which was substantiated by low RVSWI and severe echocardiographic RV dysfunction identified as two strongest predictors of need for biventricular support (28). However, this score, along with the following two (RVFRS and Drakos score) were evaluated in a smaller European study, involving 59 LVAD implantations (33). Authors demonstrated failure to predict the need for RV support in their cohort with all three proposed risk scores (33).

One of first RVF predictor models to include both pulsatile and continuous flow LVADs was RVFRS (22). It was generated based on analysis of 197 LVADs with RVF incidence of 35%, defined as a need for post-operative intravenous inotrope support for >14 days, inhaled nitric oxide for >48 h, right-sided circulatory support, or hospital discharge on an inotrope. After analysis of commonly collected preoperative clinical, laboratory, hemodynamic, and echocardiographic variables, the authors identified predictors for RVF following LVAD; need for vasopressor, AST>80 IU/L, bilirubin >2.0 mg/dL, and creatinine >2.3mg/dL and developed RVFRS as a sum of points awarded for presence of each of the 4 pre-operative variables (vasopressor requirement – 4 points, AST>80 IU/L – 2 points, bilirubin >2.0 mg/dL – 2.5 points and creatinine >2.3mg/dL – 3 points) (22). When applied to study sample, the score had 88% specificity, 35% sensitivity, 80% positive predictive value (PPV) and 80% negative predictive value (NPV). Compared to previously reported predictor models, this score had good C-statistic of 0.73 (95% confidence interval [CI] 0.65-0.81).

Another study including both pulsatile and continuous flow LVADs (27) provided multivariate analysis involving demographic, hemodynamic, laboratory, and echocardiographic variables from 175 patients with endpoint of RVF with incidence of 44%, and RVF defined as a need for inhaled nitric oxide for ≥48 hours or intravenous inotropes for >14 days and/or RVAD implantation. Score was developed as a sum of points assigned to each preoperative variable identified by multivariate model including preoperative need for intraaortic balloon pump (IABP), increased pulmonary vascular resistance (PVR), inotrope dependency, obesity, DT, angiotensin-converting enzyme inhibitor/angiotensin receptor blocker (ACE/ARB), and beta-blocker (27). The risk score was broken into 4 categories, and the C-statistic for the score was 0.743 ± 0.037. Authors also compared this score to previously described RVFRS by applying that risk score’s point values to their study population and found the C-statistic for RVFRS of only 0.61 ± 0.04 (0.73 reported in RVFRS study).

A study from University of Pittsburg (46) was the only one employing different methodology: a decision tree algorithm instead of multivariate analysis to identify predictors. It has been suggested that this prognostic tool might be superior to multivariate logistic regression with high classification accuracy and simple and intuitive representation of gathered data. This study involved 183 patients who received both pulsatile and continuous LVADs with endpoint of RVAD implantation as surrogate measure for RV failure occurring in 44% of patients. Final decision tree model comprised o8 preoperative variables; transpulmonary gradient (TPG), age, right atrial pressure (RAP), INR, heart rate, white blood count (WBC), alanine aminotransferase (ALT), and number of inotropic agents (46). This type of analysis seems to be more easily interpreted and might be useful in determining synergistic, nonlinear interactions among postoperative variables. It should be investigated and validated further, preferably including larger patient population.

Given poor predictive value of DTRS in patients with continuous flow LVAD, a study involving 1122 patients from a large multicenter clinical trial data set receiving HeartMate II LVADs for both DT and BTT was performed and identified significant predictors of 90-day mortality including older age, lower albumin, higher INR, and center volume <15 (47). As indicated, the study endpoint was defined as 90 day mortality and the incidence was 13%. Patients were divided into derivation (DC) and validation (VC) cohort. A weighted HeartMate II risk score (HMRS) was developed and patients were stratified in low-, medium-, or high-risk groups. Reported mortality was significantly different in DC low, medium, and high HMRS groups (4%, 16%, and 29%, respectively). Corresponding mortality in VC was also significantly different between groups (8%, 11%, and 25%, respectively). Reported C-statistic was 0.71 (95% CI 0.66-0.75) and it was compared to end stage liver disease (MELD) score and DTRS for predicting 90-day mortality, with corresponding C-statistic 0.66 (95% CI 0.61-0.70) and 0.6 (95% CI 0.50-0.65), respectively.

Study from University of Pennsylvania involving 218 patients (48) receiving continuous flow LVADs found 5 preoperative predictors for RV failure: central venous pressure >15 mm Hg (OR 2.0, “C”), severe RV dysfunction (OR 3.7, “R”), preoperative intubation (OR 4.3, “I”), severe tricuspid regurgitation (OR 4.1, “T”), and heart rate >100 (OR 2.0, Tachycardia - “T”). A quantitative preoperative risk score – CRITT was developed without weighing variables based on their respective odds ratios by assigning score of 0 or 1 depending on presence or absence of respective variables (48). Based on the model, authors suggested an isolated LVAD for a score of 0 or 1 and BIVAD for scores of 4 or 5. Those with scores 2 and 3 represent patients who might tolerate isolated LVAD with likely need for temporary medical or RVAD support as clinically indicated. Authors reported C-statistic of 0.80 ± 0.04, sensitivity of 87%, specificity of 75%, and NPV of 93% (48).

With improvements in ultrasound technology, there have been attempts to include echocardiographic variables in predictor models. It has been suggested that increased right to left ventricle diameter ratio is a strong predictor of RVF after LVAD, based on prospective TEE preoperative measurements (17). This finding was confirmed in another study based on analysis of patients implanted with continuous flow LVADs that included 26 TTE parameters and also adjusted for previously identified clinical predictors (49).

Other echocardiographic variables that have been identified as significant predictors of survival or RVF following LVAD include tricuspid dilatation, tricuspid incompetence, small left ventricular end diastolic diameter (LVEDD<63 mm), and early systolic equalization of RV and right atrial pressure demonstrated as decreased time interval between onset and cessation of tricuspid regurgitation flow corrected for heart rate (TRDc) (17,24,30).

In summary, there has been a lot of effort to identify risk factors for RVF following LVAD and develop clinically useful predictor scores to help improve overall survival. However, most models and respective scores faced limitations making their implementation and clinical usefulness questionable. Regardless, value of these studies lies in providing information on potential key predictors for RVF that can be taken into account in clinical decision making. Further investigation of current predictors and existing scores, as well as new studies involving larger patient populations and more sophisticated statistical prediction models, are necessary.

## Our institutional approach for minimizing risk for right ventricular failure after LVAD

It has been suggested that central venous pressure (CVP) is not a reliable surrogate of intravascular volume status. Nonetheless, elevated CVP has been identified as one of the significant predictors of RVF after LVAD, and it is still used in clinical decision making. Empirical value that we use as a target in our practice with LVAD patients is CVP<15 mm Hg. Aggressive intravenous fluid administration in a setting of already distended RV with decreased contractile capability will not increase RV output. On the contrary, it will lead to further increase in CVP, hepatic and systemic congestion, and reduced transmyocardial perfusion pressure gradient. Therefore, our approach is to be judicious with intravenous (iv) fluid administration, and rely on increasing inotropic support (milrinone, dobutamine) to help RV contractility. Also, decrease in pulmonary afterload is achieved with adequate ventilation (to avoid hypoxic pulmonary vasoconstriction) and/or use of inhaled Flolan (epoprostenol sodium) and/or NO (nitric oxide). Maintaining continuous perfusion pressure >70 mm Hg to preserve RV function and transmyocardial gradient is imperative, and often requires use of one or more vasopressors (norepinephrine, vasopressin). IABP is sometimes required to optimize RV function in coronary artery disease patients by increasing perfusion of coronary arteries.

Importance of interdisciplinary approach and excellent communication between all team members involved in perioperative management (surgeon, anesthesiologist, critical care and nursing staff) cannot be overemphasized. Transesophageal echocardiogram (TEE) surveillance for adequate ventricular filling and optimal midline septal position is important to anticipate, treat and prevent a suction event. In case of the suction event, commonly employed interventions include maintaining perfusion pressure >70 mm Hg, and careful challenging with iv fluids and temporarily allowed increase in CVP>15 mm Hg. In the event of persistently inadequate LVAD filling despite higher CVP (15-20 mm Hg), maximal inotropic and vasopressor support, and adequate respiratory function (absence of acidosis, Pao_2_>110, Paco_2_<40), RV mechanical support is commonly the next step in patient management.
