# Supplementary material for: Predictors of right ventricular failure after left ventricular assist device implantation
Source: Croat Med J. 2014 Dec;55(6):587–95. doi: 10.3325/cmj.2014.55.587 (PMC4295071; doi:10.3325/cmj.2014.55.587)
Supplement: Supplementary Table 1 [file CroatMedJ_55_s001.pdf]

Supplementary Table 1. Summary of studies identifying predictors for Right Ventricular Failure following Left Ventricular Assist Device Implantation

Authors	Title	Year	Score	Cohort	Predictive variables
Lietz et al.[44]	Outcomes of left ventricular assist device implantation as destination therapy in the post-REMATCH era: implications for patient selection.	2007	Destination Therapy Risk Score (DTRS)	N=222 patients receiving pulsatile flow LVADs for DT	Platelet count ≤ 148 x 10^3/mcg/L<br>Serum albumin ≤ 3.3 g/dL<br>INR > 1.1<br>Vasodilator therapy at time of implantation<br>Mean PAP ≤ 25.3 mm Hg,<br>AST > 45 U/dL,<br>Hematocrit < 34%,<br>Blood urea nitrogen > 51 U/dL,<br>Lack of intravenous inotropic support
Fitzpatrick et al.[28]	Risk score derived from pre-operative data analysis predicts the need for biventricular mechanical circulatory support.	2008	University of Pennsylvania Score	N=266 patients pulsatile flow LVAD which patients would require BiVAD	RVSWI≤ 0.25 mm Hg x L/m^2<br>Severe pre-operative RV dysfunction<br>Pre-operative creatinine ≥ 1.9 mg/dl<br>Previous cardiac surgery<br>SBP ≤ 96 mm Hg
Matthews et al.[22]	The right ventricular failure risk score a pre-operative tool for assessing the risk of right ventricular failure in left ventricular assist device candidates.	2008	Right Ventricular Failure Risk Score (RVFRS)	N=197 pulsatile and continuous flow LVADs	AST> 80 IU/L<br>Bilirubin > 2.0 mg/dL<br>Creatinine > 2.3mg/dL need for vasopressor
Drakos et al.[27]	Risk factors predictive of right ventricular failure after left ventricular assist device implantation.	2010	University of Utah Risk Score	N=175 patients pulsatile and continuous flow LVADs	Need for IABP<br>Increased PVR<br>Inotrope dependency<br>Obesity<br>Destination Therapy<br>ACE/ARB<br>Beta-blocker
Wang et al.[46]	Decision tree for adjuvant right ventricular support in patients receiving a left ventricular assist device.	2012	University of Pittsburg Risk Score	N=183 patients who received both pulsatile and continuous LVADs	Transpulmonary gradient<br>Age<br>Right atrial pressure<br>INR<br>WBC<br>ALT<br>Heart rate<br>Number of inotropic agents
Cowger et al.[47]	Predicting survival in patients receiving continuous flow left ventricular assist devices: the HeartMate II risk score.	2013	HeartMate II Risk Score (HMRS)	N=1122 patients receiving HeartMate II LVAD	Mortality predictors:<br>Older age<br>Lower albumin<br>Higher INR<br>Center volume < 15
Atluri et al.[48]	Predicting right ventricular failure in the modern, continuous flow left ventricular assist device era.	2013	CRITT score	N=218 patients receiving continuous flow LVADs	Central venous pressure >15 mmHg<br>Severe RV dysfunction<br>Preoperative intubation severe tricuspid regurgitation<br>Heart rate >100
