# Supplementary material for: Predictors of right ventricular failure after left ventricular assist device implantation
Source: Croat Med J. 2014 Dec;55(6):587–95. doi: 10.3325/cmj.2014.55.587 (PMC4295071; doi:10.3325/cmj.2014.55.587)
Supplement: Supplementary Table 2 [file CroatMedJ_55_s002.pdf]

**Supplementary Table 2. Summary of studies using preoperative echocardiographic variables to predict Right Ventricular Failure following Left Ventricular Assist Device Implantation**

| Authors | Title | Year | Cohort | Predictive variables |
|---|---|---|---|---|
| **Potapov et al.**[24] | Tricuspid incompetence and geometry of the right ventricle as predictors of right ventricular function after implantation of a left ventricular assist device. | 2008 | N=54 | Tricuspid incompetence and RV geometry |
| **Topilsky et al.**[30] | Echocardiographic predictors of adverse outcomes after continuous left ventricular assist device implantation. | 2011 | N=83 | Small left ventricular end diastolic diameter (LVEDD <63 mm)<br>Early systolic equalization of RV and right atrial pressure demonstrated as decreased time interval between onset and cessation of tricuspid regurgitation flow corrected for heart rate (TRDc). |
| **Kukucka et al.**[17] | Right-to-left ventricular end-diastolic diameter ratio and prediction of right ventricular failure with continuous-flow left ventricular assist devices. | 2011 | N=135 | RV/LV end-diastolic diameter ratio > 0.72 |
| **Vivo et al.**[49] | Increased right-to-left ventricle diameter ratio is a strong predictor of right ventricular failure after left ventricular assist device. | 2013 | N=109 | RV/LV diameter ratio $\geq 0.75$ |
